# Computerised cognitive training combined with nutritional counseling in mild cognitive impairment - results of a 2 × 2 factorial randomized controlled trial (BrainFit-nutrition study)

**DOI:** 10.1186/s13195-026-02255-6

**Published:** 2026-11-09

**Authors:** Julia-Sophia Scheuermann, Elmar Graessel, Etienne Hanslian, Julia Schiele, Melanie Dell’oro, Martin Schmitt, Michael Jeitler, Christian S. Kessler

**Affiliations:** 1 Section of Medical Psychology and Medical Sociology, Department of Psychiatry and Psychotherapy, Uniklinikum Erlangen, Friedrich-Alexander-Universität Erlangen-Nürnberg (FAU), Schwabachanlage 6, 91054 Erlangen, Germany; 2 Center for Health Services Research in Medicine, Department of Psychiatry and Psychotherapy, Uniklinikum Erlangen, Friedrich-Alexander-Universität Erlangen-Nürnberg (FAU), Schwabachanlage 6, 91054 Erlangen, Germany; 3 Department for Dementia Research and Nursing Science, University for Continuing Education Krems, Krems, Austria; 4 Department of Internal Medicine and Nature-Based Therapies, Immanuel Hospital Berlin, 14109 Berlin, Germany; 5 Institute of Social Medicine, Epidemiology and Health Economics, Charité – University Medical Center Berlin, Corporate Member of Freie Universitat Berlin, and Humboldt-Universitat Zu Berlin, Berlin, Germany; 6 Charité Competence Center for Traditional and Integrative Medicine (CCCTIM), Charité - University Medical Center Berlin, Corporate Member of Freie Universität, Berlin and Humboldt-Universität zu Berlin, Berlin, Germany; 7 Department of Integrative Health Care and Prevention, University of Augsburg & University Hospital Augsburg, Augsburg, Germany; 8 Institute for General Practice and Interprofessional Care, University Hospital Tuebingen, Tuebingen, Germany; 9 Robert Bosch Center for Integrative Medicine and Health, Bosch Health Campus, Stuttgart, Germany

**Keywords:** Mild cognitive impairment, MCI, Computerized cognitive training, Nutritional counseling, Whole-food plant-based nutrition, Non-pharmacological intervention, MoCA, Randomized controlled trial

## Abstract

**Background:**

Computerized cognitive training (CCT) and nutritional interventions may improve cognitive functioning in individuals with mild cognitive impairment (MCI). This study investigated whether a combination of CCT and online nutritional counseling can improve global cognition in individuals with MCI. We also evaluated differences between MCI-specified and active control versions of the interventions.

**Methods:**

In this 2 × 2 factorial randomized controlled trial (BrainFit-Nutrition study), conducted fully remotely, participants in Germany with a psychometric diagnosis of MCI (age 60+) were randomized into four groups (Intention to treat, *n* = 270). For each intervention, one MCI specified and one active control version were developed (cognition: individualized CCT (iCCT) vs. basic CCT (bCCT); nutrition: whole-food plant-based nutrition (WFPB) vs. German Nutrition Society (DGE)). At baseline and after six months, cognitive functioning was assessed with the Montreal Cognitive Assessment (MoCA; primary outcome: change after six months). A mixed-model ANCOVA (t0-t6, complete case strategy (CC), *n* = 228) was applied for the main analysis.

**Results:**

All four groups achieved a significant improvement of approximately 2 points on the MoCA after six months. Estimated marginal means varied from *M* = 2.44 (iCCTxDGE) to *M* = 1.77 (iCCTxWFPB), notably without significant group differences. The 2 × 2 mixed ANCOVA revealed a significant main effect of time (*F* = 3.96, *p* = 0.048, partial η^2^ = 0.018). The specific content of the respective modalities being compared (iCCT vs. bCCT; WFPB vs. DGE-oriented counseling) did not appear to make a relevant group difference with regard to the primary outcome. After 6 months, 55% of all participants reached non-MCI values on the MoCA.

**Conclusion:**

The results of this study suggest, that in people with MCI, notable improvement in global cognition can be achieved in a six-months intervention period. When CCT is combined with digital nutritional counseling, the form of intervention – whether elaborate or basic – apparently did not modify the outcome; rather, this data set suggests that it is worthwhile to proactively undertake something in both domains. Future clinical studies in mixed-methods-designs, including usual-care comparators, basic science study modules, qualitative aspects and health economic variables are warranted to gain a better understanding of mechanisms, sustainability of effects, clinical relevance, and cost-effectiveness.

**Trial registration:**

ISRCTN10560738; registered 11/23/2021.

**Supplementary Information:**

The online version contains supplementary material available at https://doi.org/10.1186/s13195-026-02255-6.

## Background

Before persons’ health condition progress to dementia, they often suffer from mild cognitive impairment (MCI) [1, 2]. Therefore, MCI is likely a crucial period for intervention to avoid conversion to dementia. Since available pharmacological treatments for early Alzheimer’s disease show standardized mean differences below 0.25 over a treatment period of at least one year, non-pharmacological treatment options are of clinical interest [3, 4]. There are well-established medical and psychosocial risk factors for dementia [5], offering starting points for dementia prevention [6, 7], including cognitive training or cognitive stimulation [8, 9], physical exercise [10–12], and an anti-inflammatory diet [13–16]. A combination of such approaches as a multicomponent intervention may also be beneficial for individuals suffering from cognitive impairment [17–21].

Cognitive training is recommended as a non-pharmacological intervention due to its beneficial effects, especially in early stages of dementia, such as the MCI stage [22]. Cognitive training stimulates neuroplasticity, task-dependent brain activation, and brain metabolism [9]. Thus, cognitive training may prevent or delay the onset of dementia [22, 23]. In the current decade, cognitive training has increasingly been performed digitally [9, 23, 24], enabling barrier-reduced access and thereby offering an effective alternative to analogous exercises [25, 26]. Computerized cognitive training (CCT) comprises computer-based tasks that include cognitive exercises, games, and virtual reality with real-time feedback [27] and is well-accepted among people with MCI [27, 28]. CCT can implement adaptive learning systems for custom-tailored levels of task difficulty, with positive effects on different outcomes [9, 24]. In addition, well-designed game elements and higher gamification levels may increase effectiveness, adherence, and motivation for optimized training success likelihoods [29]. Meta-analyses have shown a small but significant positive effect of CCT on global cognition and specific cognitive functions compared with control groups (CGs), especially when compared with passive CGs [23, 26, 29]. A relative lack of high-quality studies in people with MCI still poses a substantial limitation [26, 30], indicating a need for optimized intervention doses and adapted, individualized programs.

In a previous study we developed an individualized CCT (iCCT), specialized for people with MCI [31, 32]. The iCCT integrated various cognitive parameters—e.g., information processing speed, short-term memory, and decision making—in a playful manner and AI-based dynamically adapted task difficulty. As a counterpart, we constructed the basic CCT (bCCT), which is characterized by fewer exercises, simpler exercises, and no use of AI. The results of the MCI-CCT study showed that using iCCT or bCCT three times a week for about 30 min led to significant improvement within six months in global cognition (MoCA score) in people with MCI living at home [32], with a significantly greater increase in the iCCT group. The BrainFit-Nutrition study (BF-N) extended this study design by including an additional intervention: online nutritional counseling.

Healthy dietary patterns are associated with preserved cognitive function [13, 16, 33–36]. Diets such as the Mediterranean Diet (MedDiet), the MIND Diet (Mediterranean-DASH Intervention for Neurodegenerative Delay), and the DASH Diet (Dietary Approaches to Stop Hypertension) emphasize minimally processed plant foods and have been linked to reduced inflammation and a lower risk of cognitive decline and dementia [15, 37–40]. A whole-food plant-based (WFPB) diet represents a more challenging dietary approach compared with such diets due to its emphasis on minimally processed plant-based foods and the restriction of animal products. Although evidence for the effectiveness of the Mediterranean and MIND diets exists, there are still limited results from controlled studies specifically examining WFPB interventions for MCI.

Importantly, WFPB differs from Mediterranean, MIND, and DASH dietary patterns in that it excludes or strongly restricts animal-derived products rather than primarily moderating their intake. Although Mediterranean and MIND dietary patterns currently have a stronger evidence base for cognitive outcomes in older adults, WFPB counseling was chosen as the MCI-specified nutritional intervention based on the hypothesis that a more pronounced dietary shift might produce stronger mechanistic effects. The potential cognitive benefits of WFPB nutritional patterns may be attributed to several interacting pathways, including reduction of vascular and metabolic risks such as hypertension, diabetes, hyperlipidemia, obesity, and cardiovascular disease [41–47], anti-inflammatory and antioxidant effects through reduced intake of saturated animal fats, heme iron, processed meat, and animal proteins combined with higher intake of fiber, polyphenols, and phytochemicals [48–55], and neurobiological mechanisms such as improved endothelial function, cerebrovascular perfusion, and insulin signaling in the brain [41, 51–55]. Thus, WFPB was used as a more focused nutritional contrast to DGE-oriented healthy-diet counseling, while the latter represented an active, evidence-based healthy omnivorous comparator rather than a passive or usual-diet control.

Clinical interventions indicate that plant-based diets may improve diabetes outcomes [42, 43], coronary heart disease [44, 45], and overweight, and even modest weight reduction has been associated with better attention and memory performance [46, 47]. Reduced consumption of animal proteins [48], alongside high intake of dietary fiber and phytochemicals/-nutrients [49, 50], may contribute via anti-inflammatory processes. Since MCI has also been linked to microinflammatory processes [51], bioactive compounds derived from plants, fungi, and algae may be particularly relevant [52–55]. A recent randomized pilot trial of a comprehensive multidomain lifestyle intervention comprising a WFPB diet, moderate aerobic exercise, stress management, and enhanced social support reported improvements in cognition in individuals with MCI or early Alzheimer’s disease [56]. It should be noted that this trial tested a combined lifestyle program, not a WFPB dietary intervention in isolation. Its findings therefore constitute evidence for multicomponent lifestyle change that includes WFPB, rather than standalone dietary evidence [56].

Thus, both CCT and nutrition may improve the cognitive capacities of people with MCI. Thus, in the BF-N study, we developed one MCI-specified version of the intervention for each component (iCCT & online counseling focusing on a WFPB diet) and corresponding active controls (bCCT & online counseling focusing on a diet aligned with recommendations for healthy nutrition by the German Nutrition Society (in German: Deutsche Gesellschaft für Ernährung, DGE). Previous papers from this trial cohort have included baseline analyses of daily activities [57], depression [58], the risk factor of alcohol consumption [59], as well as follow-up data on user experiences with CCTs [60] and longitudinal data on dietary behavior and adherence data [61].

### Aim of the study

The primary aim of this 2 × 2 randomized controlled trial (RCT) was to test whether the MCI-specified versions of the cognitive and nutritional interventions produced greater improvement in the cognitive abilities, measured with the Montreal Cognitive Assessment (MoCA) than their active control versions, and whether the combination of the MCI-specified versions resulted in an additional interaction effect in individuals with MCI over a six-months intervention period [62]. We considered three study hypotheses:

Primary hypothesis I: iCCT leads to statistically significantly greater improvements in global cognition during the six-months intervention period compared with bCCT.

Primary hypothesis II: Online nutritional group counseling focusing on a WFPB diet leads to statistically significantly greater improvements in global cognition during the six-months intervention period compared with online nutritional group counseling focusing on a healthy diet recommended by the DGE.

Secondary hypothesis: iCCT in combination with online nutritional group counseling, focusing on a WFPB diet, has a positive interaction effect. The combination of these two versions of the interventions will show more improvement in global cognition than all other groups during the six-months intervention period in people with MCI.

## Methods

### Study design

The BF-N study was a prospective 2 × 2 factorial RCT that was conducted completely remotely [62]. Each intervention comprised an MCI specified version of the intervention and an active control version. The study included people living in Germany with psychometrically diagnosed MCI. The first participant entered the study on 01/25/2022, and the last patient completed t6 on 05/09/2023. The intervention period was six months, with a follow-up after another six months (t12). The data were collected at baseline (t0) and directly after the intervention period (t6) as well as after 12 months (t12). However, the current analysis focused on the pre-post intervention results (t0-t6), follow-up data will be reported elsewhere.

All procedures were approved by the Ethics Committee of the Friedrich-Alexander-Universität Erlangen-Nürnberg (Ref. 21–318_1-B). Participation was voluntary, and participants were free to leave the study at any time for any reason. Written informed consent was obtained from all participants. The study was prospectively registered on 11/23/2021 at ISRCTN registry (Trial identification number: ISRCTN10560738; https://doi.org/10.1186/ISRCTN10560738). Further details on the study design are available in the study protocol [62].

### Recruitment

The a priori sample size calculation indicated a sample size of *n* = 200, evenly distributed across the four groups, to achieve a power of *f* ≥ 0.24, taking into account α = 0.05, β = 0.20, a correlation between repeated measures of 0.5, and a non-sphericity correction of 1 [62]. Various methods were used to recruit study participants and to fulfill the calculated sample size: In addition to advertising by two German health insurance companies, we initiated social media activities on Facebook and advertised the study in the print editions and websites of various magazines and newsletters. Furthermore, memory clinics throughout Germany were included in the recruitment process. In addition, Bavarian pharmacies were made aware of the study in two newsletters in collaboration with the Bavarian State Chamber of Pharmacists (Bayerische Landesapothekerkammer). Furthermore, the study was advertised in Berlin as part of advertisements for public transportation alongside with other research projects by the Berlin working group.

### Eligibility of participants

Participants had to meet several inclusion criteria to participate in the BF-N study. During the online-screening, interested participants were screened for basic cognitive functions, including a personal consultation regarding the results of the screening. Individuals who met the inclusion criteria were informed about the eligibility of study participation and invited to participate [62].

Inclusion criteria were: (1) MCI, psychometrically operationalized by a global cognition score on the Montreal Cognitive Assessment (MoCA) ≤24 (cut-off for cognitive impairment) and at the same time a score on the Mini-Mental State Examination (MMSE) ≥24 (cut-off for no dementia), (2) possession of a computer (Windows/Linux/MacOS) with microphone and camera, laptop, or tablet with access to the internet and basic skills in their use, (3) age ≥ 60, and (4) informed consent. Exclusion criteria were: (1) completely blind or deaf, (2) no personal computer, laptop, or tablet with access to the internet, (3) normal cognition, operationalized by a score of global cognition on the MoCA > 24, (4) dementia, operationalized by a score on the MMSE < 24, (5) acute depression, operationalized by a score on the 9-item Patient Health Questionnaire (PHQ-9) ≥12, or (6) other psychiatric or neurologically diagnosed diseases (checklist) [62].

### Randomization and blinding

After completed screening process and written informed consent, participants were randomly assigned to one of the four intervention groups. Randomization was performed externally by the Institut für Medizininformatik, Biometrie und Epidemiologie (IMBE) of the Friedrich-Alexander-Universität Erlangen-Nürnberg. The statistician was blinded to the groups. Due to the rolling recruitment, we performed a block randomization to one of the four study groups, stratified by gender, age, and baseline global cognition (MoCA), with variable block sizes [62]. People from the same household were assigned to the same group. After randomization, the IMBE transmitted the final group allocation to the study headquarters. Participants were then provided with an access link and a serial number for the CCT program based on their group assignment (iCCT vs. bCCT). Participants’ assignment to the respective intervention group was masked by using non-specific wording (e.g., “brain food” and “cognitive training”). The student assistants conducting the outcome assessment were blinded to participants’ group allocation at all times. Before the last randomization block, we checked that the randomization balance was adequate.

### Interventions

#### Type I: Cognition with computerized cognitive training

The two computerized applications (intervention and active control) were available for Windows, MacOS, and Linux PC/laptop and Android tablet. More information on the CCT and users’ experiences with it have already been published [60, 62].

##### Version Ia: iCCT for people with MCI

The ten game-based exercises (e.g. applying rules, pattern recognition) included in this training application were selected to meet the performance levels expected from people with MCI based on an adaptive difficulty level. The iCCT address the basic parameters of information processing and short-term memory and require different types of decision making. Using machine learning, each participant is provided with exercises tailored to their individual skill level. Detailed information on the machine learning system are already published [62]. The application selects the highest difficulty level that the participant is likely to solve as entry level. During the training phase the machine learning system generates the ideal difficulty level of training for each participant using cognitive test parameters that are assessed individually once a month. Thus, the iCCT aims to enhance the positive effects of CCT by providing exercises at the difficulty level that is most suitable for each participant.

##### Version Ib: bCCT (active control)

The bCCT includes four exercises on the basis of quizzes and visual tasks (e.g. rotating picture quiz, quiz-show). They are designed to be “playful” and require simple strategies as well as long-term memory. The starting difficulty of the exercises is based solely on the participant’s previous success with this exercise. Machine learning methods are not implemented. The aim of the bCCT is to offer the active control group an enjoyable computerized leisure activity with a limited number of cognitive tasks. For more information see study protocol [62].

#### Type II: Nutrition via online nutritional group counseling

The nutritional intervention was implemented as a six-months online group counseling program. Sessions were conducted via a pseudonymized online conference tool. Groups of 10–25 participants attended twelve 90-minute bi-weekly counseling-sessions, each following a standardized format: a brief introduction, a structured educational component (e.g., basics of healthy eating, nutrient groups, mindful eating), practical skill-building (e.g., meal planning, virtual cooking demonstrations), and a closing segment with goal setting and homework. Courses were led by three qualified nutritionists with training in group facilitation [61]. To support adherence, participants received monthly food boxes with products consistent with their dietary recommendations, serving as an implementation strategy to facilitate transfer of counseling content into everyday practice [61].

Participants were randomized to one of two dietary counseling approaches:

##### Version IIa: Counseling focusing on a whole-food plant-based diet, WFPB

This program promoted a predominantly WFPB-pattern (vegetables, whole grains, legumes, fruits, nuts, seeds) with no energy restrictions, emphasizing foods with potential neuroprotective properties (e.g., green leafy vegetables, berries, soy products, turmeric, green tea, omega-3-rich oils). Participants were encouraged to avoid animal products and minimize highly processed foods, red and processed meats, refined grains, and sugary beverages. Importantly, the WFPB protocol differed from the Mediterranean, MIND, and DASH diets in that it excluded or strongly restricted animal-derived products rather than moderating their intake. At baseline, 95.5% of WFPB-group participants reported following an omnivorous diet [63]. The program thus entailed a substantial change in dietary habits.

##### Version IIb: Counseling focusing on a diet recommended by the German nutrition society, DGE (active control)

This program followed DGE recommendations for a balanced omnivorous diet, encouraging daily vegetables, fruits, and whole grains alongside moderate amounts of fish, poultry, lean meat, dairy, and eggs, while limiting saturated fat, added sugars, and highly processed items. The DGE-based counseling was delivered in the same format as the WFPB-arm. At baseline, 94.6% of DGE-group participants reported following an omnivorous diet [61], consistent with the program’s optimization rather than fundamental change of current eating patterns. The DGE guidelines constitute a scientifically validated, evidence-based dietary reference standard, and therefore represent an active comparator rather than an inactive control [63].

### Measures

The participants were interviewed at baseline and after the intervention phase (after six months) by videoconference. The fulfilment of the baseline data after checking the study eligibility and willingness to participate was conducted by telephone interview.

#### Primary outcome

*Montreal Cognitive Assessment* (MoCA): The MoCA is a performance test that screens for MCI and assesses global cognition status [64]. It consists of more difficult items than the MMSE and is therefore better able to detect MCI [64–67]. A score ≤ 24 (range 0–30 points) indicates cognitive impairment [68, 69]. Parallel versions of the German translation of the MoCA for videoconferencing were used in the study to eliminate learning effects: V.8.1 was administered at t0 (screening) and V.8.2 at t6.

#### Screening instruments

*Mini-Mental Status Examination* (MMSE): The MMSE is the most frequently used screening test for dementia [70], with five areas of cognitive functioning: orientation, registration, attention and calculation, recall, and language. A score ≤ 23 (range 0–30 points) indicates a dementia syndrome, whereas scores > 23 are interpreted as no dementia [71]. For this study, we used an adapted validated version of the MMSE for an audio-visual setting based on Timpano et al. [72, 73].

*Patient Health Questionnaire − 9 items* (PHQ-9): The PHQ-9 is a short self-assessment screening tool for depression, often used in primary care settings [74]. It consists of nine items covering the nine DSM-IV criteria by asking patients about their experiences during the last two weeks. The items are rated on a four-point scale ranging from 0 (not at all) to 3 (nearly every day). The total sum score indicates varying levels of depression. A cut-off ≥12 was found to show a good balance between sensitivity and specificity [75].

#### Other variables - Sociodemographic and health-related data

The student assistants used a standardized questionnaire to assess sociodemographic and health-related data as well as modifiable risk factors for MCI. Sociodemographic data were recorded at baseline and comprised information on age, sex, marital status, highest education level (as assessed with the ISCED [76]), employment status, monthly income, and household size. Furthermore, as modifiable risk factors for MCI, the status of general mental activities, physical activities, social participation, sleeping habits, average liquid intake, eating habits, alcohol consumption, nicotine consumption, and visual and hearing capacity were assessed at baseline and t6. Health-related data, which were also recorded at baseline, and—if relevant—at t6 and t12, comprised information on diseases, medication, body weight, body height, and dementia cases in the family.

To assess the effects and side effects on brain function (i.e., stimulation or sedation) of prescribed medication taken by the participants, we used the “medication score” [77]. All effects were ranked from −2 (strong sedating effect) to + 2 (strong stimulating effect) by two clinical pharmacologists at the Uniklinikum Erlangen and summed to form the medication score.

To measure the individual vascular risk level, we calculated the vascular risk score, which represents the number of risk factors consisting of smoking, hypertension, hypercholesterolaemia, and diabetes mellitus. The score ranges from 0 (no vascular risk factors) to 4 (four vascular risk factors).

To analyze the comorbidity-related mortality rate, we used the Charlson Comorbidity Index, updated and validated by Quan et al. [78]. To form the index, 12 diseases are assigned weights ranging from 1 point (e.g., chronic pulmonary disease) to 6 points (e.g., metastatic solid tumor). The sum score ranges from 0 to 24 points. Higher scores indicate a higher one-year comorbidity-related mortality rate, whereby a score of 5 is associated with an 85% one-year mortality risk.

The frequency of participation in the cognitive training program was assessed on the basis of self-reported training frequency (minutes/day), number of training days/week, and number of monthly uploads (adherence CCTB, sum score 1–7). The frequency of participation in the online nutritional counseling courses was assessed per session (nutrition adherence, sum score 0–12). For both adherence variables, the number of participants attending every nutritional counseling session or every CCTB upload was documented. Furthermore, each participant was asked by telephone about the occurrence of adverse events (AE) to assess safety. If participants reported an AE, they were asked about its intensity and progression in detail. The study center evaluated the causal relationship with the intervention, recommended actions, and whether a severe adverse event (SAE) had occurred.

### Data collection

Psychology students were trained at the study headquarters in Erlangen to collect data in semi-structured interviews with the study participants with MCI.

Data were collected at baseline (t0) and immediately after the end of the intervention (t6). The cognition-related parameters were collected approximately two weeks before t0 during the screening procedure and thus used as baseline data. The data were collected digitally by student assistants via video surveys using the certified software samedi (Samedi GmbH, paid access for video consultation licenses and a widget for appointment booking at t0 and t6) or telephone interview (t0). They were blinded to participants’ group assignments at all times. Data were collected with the RedCap software, which enabled a plausibility check of the data to be carried out directly during data collection using the programmed variable ranges. In addition, the CCTB [79] was recorded on the participants’ digital devices and voluntarily made available to the Erlangen study headquarters by the participants via digital upload to a data-secure cloud.

In addition to the sealed envelope containing the MoCA and MMSE documents, the participants received a questionnaire with sociodemographic as well as health-related questions for the examination appointments, including the PHQ-9 to record depressive symptoms.

Digital participant management was carried out by the webMODYS software (provided by Leibniz Institute for Prevention Research and Epidemiology—BIPS, Bremen, Germany), which was individually programmed for the project and hosted at Uniklinikum Erlangen.

### Statistical analysis

According to the study protocol [62], the analysis should have been conducted using intention-to-treat (ITT) and per-protocol analyses; however, this analysis strategy was modified after start of the interventions. Due to a dropout rate of 15.6% between baseline and t6 follow-up, and taking into account current developments in the statistical analysis of pharmacological RCTs on dementia prevention, which favor a modified ITT approach [80], the complete case (CC) analysis was established as the primary analysis strategy. The originally planned ITT strategy was instead conducted as a sensitivity analysis to test the robustness of the CC results against the ITT model, which contains 15.6% estimated data for the primary outcome variable. The per-protocol analysis specified in the study protocol was not performed because no operationalized adherence criteria had been defined prior to the start of the study.

Thus, the analysis sample comprised *n* = 228 cases in the main analysis (CC), and *n* = 270 people in the sensitivity analysis (ITT). To enable the ITT analysis, 42 (15.6%) missing MoCA t6 values (primary outcome: cognition) were imputed by applying the expectation maximization algorithm (E-M-algorithm) with group, MoCA score at t0, and all available t0 variables (Table 1) as predictors.

**Table 1:** Baseline characteristics of the study participants in the complete case sample (*N* = 228)

Variables	iCCT+WFPB (n = 55)	iCCT+DGE (n = 59)	bCCT+WFPB (n = 54)	bCCT+DGE (n = 60)	p	Group difference (│effect size│)	Total (n = 228)
**Sociodemographics**							
Age (years), *M* (*SD*)	70.6 (6.6)	71.0 (6.5)	70.3 (5.8)	70.9 (7.8)	0.951	0.01^$^	70.7 (6.7)
Sex (female), *n* (%)	29 (52.7)	31 (52.5)	26 (48.1)	33 (55.0)	0.907	0.05^#^	119 (52.2)
Education level (ISCED)^1^, *n* (%)					0.566	0.09^#^	
Low/middle	21 (38.2)	22 (37.3)	26 (48.1)	22 (36.7)			91 (39.9)
High	34 (61.8)	37 (62.7)	28 (51.9)	38 (63.3)			137 (60.1)
Household net income per month (Euro), *M* (*SD*)	2646 (702)	2453 (815)	2625 (720)	2690 (623)	0.302	0.02^$^	2602 (720)
Employment (yes), *n* (%)	17 (30.1)	16 (27.2)	14 (25.9)	17 (28.3)	0.946	0.04^#^	64 (28.1)
Other people living in the household (yes), *n* (%)	39 (70.9)	39 (66.1)	42 (77.8)	38 (63.3)	0.365	0.12^#^	158 (69.3)
MoCA Baseline, *M* (*SD*)	22.27 (1.55)	22.32 (1.60)	22.35 (1.62)	22.28 (1.47)	0.993	0.01^$^	22.31 (1.55)
MoCA t6, *M* (*SD*)	24.11 (3.17)	24.68 (2.54)	24.43 (2.57)	24.35 (2.81)	0.749	0.01^$^	24.39 (2.77)
**Risk factors for cognitive decline**							
Depressiveness (PHQ-9^2^), *M* (*SD*)	3.7 (2.8)	3.9 (2.8)	3.7 (2.7)	4.0 (3.2)	0.923	0.01^$^	3.8 (2.9)
Vascular risk (sum score^3^), *M* (*SD*)	1.0 (0.82)	1.13 (0.87)	1.04 (0.92)	1.12 (0.90)	0.818	0.01^$^	1.08 (0.87)
Smoking (last 30 days, yes), *n* (%)	5 (9.1)	1 (1.7)	2 (3.7)	3 (5.0)	0.421	0.12^#^	11 (4.8)
Hypertension (yes), *n* (%)	23 (41.8)	29 (49.2)	20 (37.0)	31 (51.7)	0.378	0.12^#^	103 (45.2)
Hypercholesterolaemia (yes), *n* (%)	24 (43.6)	30 (50.8)	27 (50.0)	26 (43.3)	0.772	0.07^#^	107 (46.9)
Diabetes (yes), *n* (%)	3 (5.5)	7 (11.9)	6 (11.1)	7 (11.7)	0.617	0.09^#^	23 (10.1)
Medication score^4^, *M* (*SD*)	−0.05 (0.3)	−0.07 (0.25)	−0.11 (0.57)	−0.08 (0.33)	0.880	0.01^$^	−0.08 (0.38)
Cognitive activities (hours per week), *M* (*SD*)	15.6 (9.9)	17.9 (9.4)	17.5 (12.8)	21.6 (22.1)	0.159	0.03^$^	18.2 (14.7)
Physical activities (hours per week), *M* (*SD*)	9.8 (6.4)	11.8 (9.9)	10.5 (8.9)	10.7 (8.3)	0.649	0.01^$^	10.7 (8.5)
Social activities (hours per week), *M* (*SD*)	8.8 (6.6)	11.6 (9.9)	9.0 (5.5)	10.9 (8.4)	0.148	0.02^$^	10.1 (8.0)
Charlson Index^5^, *M* (*SD*)	0.16 (0.42)	0.34 (0.73)	0.37 (0.73)	0.37 (0.74)	0.314	0.02^$^	0.31 (0.67)
Body Mass Index, normal (≥19 and ≤25)^6^ (yes), *n* (%)	49 (89.1)	52 (88.1)	47 (87.0)	53 (88.3)	0.990	0.02^#^	201 (88.2)
**Interventions**							
Adherence to nutritional counseling^7^, *M* (*SD*)	7.2 (3.7)	7.6 (3.9)	7.6 (3.5)	7.4 (3.8)	0.916	0.01^$^	7.4 (3.7)
Full adherence to nutritional counseling^8^, *n* (%)	11 (20.0)	13 (22.0)	13 (24.1)	11 (18.3)	0.889	0.05^#^	48 (21.1)
Adherence to CCTB (months) ^9^, *M* (*SD*)	5.5 (2.3)	5.8 (2.1)	5.9 (1.9)	5.7 (2.0)	0.624	0.01^$^	5.7 (2.1)
Full adherence to CCTB^8^, *n* (%)	38 (69.1)	46 (78.0)	45 (83.3)	46 (76.7)	0.366	0.12^#^	175 (76.8)

*Note*. ^$^ η^2^; ^#^ Cramer’s V; iCCT: individualized computerized cognitive training; bCCT: basic computerized cognitive training; WFPB: whole-food plant-based nutritional counseling; DGE: nutritional counseling as recommended by the German Nutrition Society; ^1^ In the low education category, there were only *n* = 7 cases in three groups as determined by the ISCED (International Standard Classification of Education), so these cases were combined with the medium ISCED category; ^2^ Patient Health Questionnaire 9; ^3^ Number of vascular risk factors: range 0 (no vascular risk factor) to 4 (four vascular risk factors); ^4^ Sedating (<0) or stimulating (>0) effect or side effect of the prescribed medications: sum score for each medication, which was ranked from −2 (strong sedating effect) to + 2 (strong stimulating effect); ^5^ Updated Charlson Comorbidity Index: range: 0–24; ^6^ normal range of the Body Mass Index; ^7^ adherence captured through the number of counseling sessions attended; ^8^number of participants attending each nutritional counseling session or every CCTB (Computerized Cognitive Test Battery [79]), upload; ^9^ number of months with active upload of the CCTB

In the first part of the statistical analysis framework, the characteristics of the sample and group differences are described using inferential statistical procedures and their effect sizes: Cramer’s *V* (crosstabs), Cohen’s *d* (*t*-test), and η^2^ (ANOVA/ANCOVA). The interpretation of the effect sizes and the decision about which variables to include were based on Cohen’s guidelines [81]: Cramer’s *V* with limits of 0.1 (low), 0.3 (medium), and 0.5 (high); Cohen’s *d* with limits of 0.2 (low), 0.5 (medium), and 0.8 (high); and η^2^ with limits of 0.010 (low), 0.059 (medium), and 0.138 (high).

The inferential statistical procedures we used were χ^2^-tests, *t*-tests, and 2 × 2 ANCOVAs. Differences between the groups in the CC and ITT samples were analyzed for categorically scaled variables with the χ2-test and for continuous variables with ANOVAs. The change in the global cognition score between t0 and t6 was analyzed with a *t*-test for dependent samples in the total sample as well as in the four intervention groups (iCCT + WFPB, iCCT + DGE, bCCT + WFPB, and bCCT + DGE).

The main analysis for testing the three hypotheses (two primary and one secondary hypothesis) was conducted with a 2 × 2 mixed ANCOVA for the dependent variable global cognition (MoCA) using the within-subject factor time (t0, t6) and the between-subject factor group (Intervention Type I: cognitive intervention iCCT vs. bCCT and Intervention Type II: nutrition intervention WFPB vs. DGE). Furthermore, the model was adjusted for relevant baseline variables as covariates. Variables were included as relevant covariates if they had an evidence-based [82] preventive influence on the pathological decline of cognition and showed a relevant effect (>small effect size) of the group difference in the study sample. To rule out multicollinearity, the covariates were allowed to correlate with each other with a maximum of *p* = 0.80 [83, 84]. Within a sensitivity analysis, the main analysis was also conducted for the ITT sample (I). The procedure was the same as for the main analysis.

Furthermore, we assessed the reversion rate from MCI (MoCA score ≤ 24) to normal cognition (MoCA > 24, MCI criterium psychometrically not fulfilled) in the primary outcome (MoCA score) as well as the occurrence of AEs on a descriptive level. The reversion rate for individuals at the cutoff threshold to cognitive normal (MoCA 22–24) was examined separately.

## Results

### Screening and participant flow

**Fig. 1:**
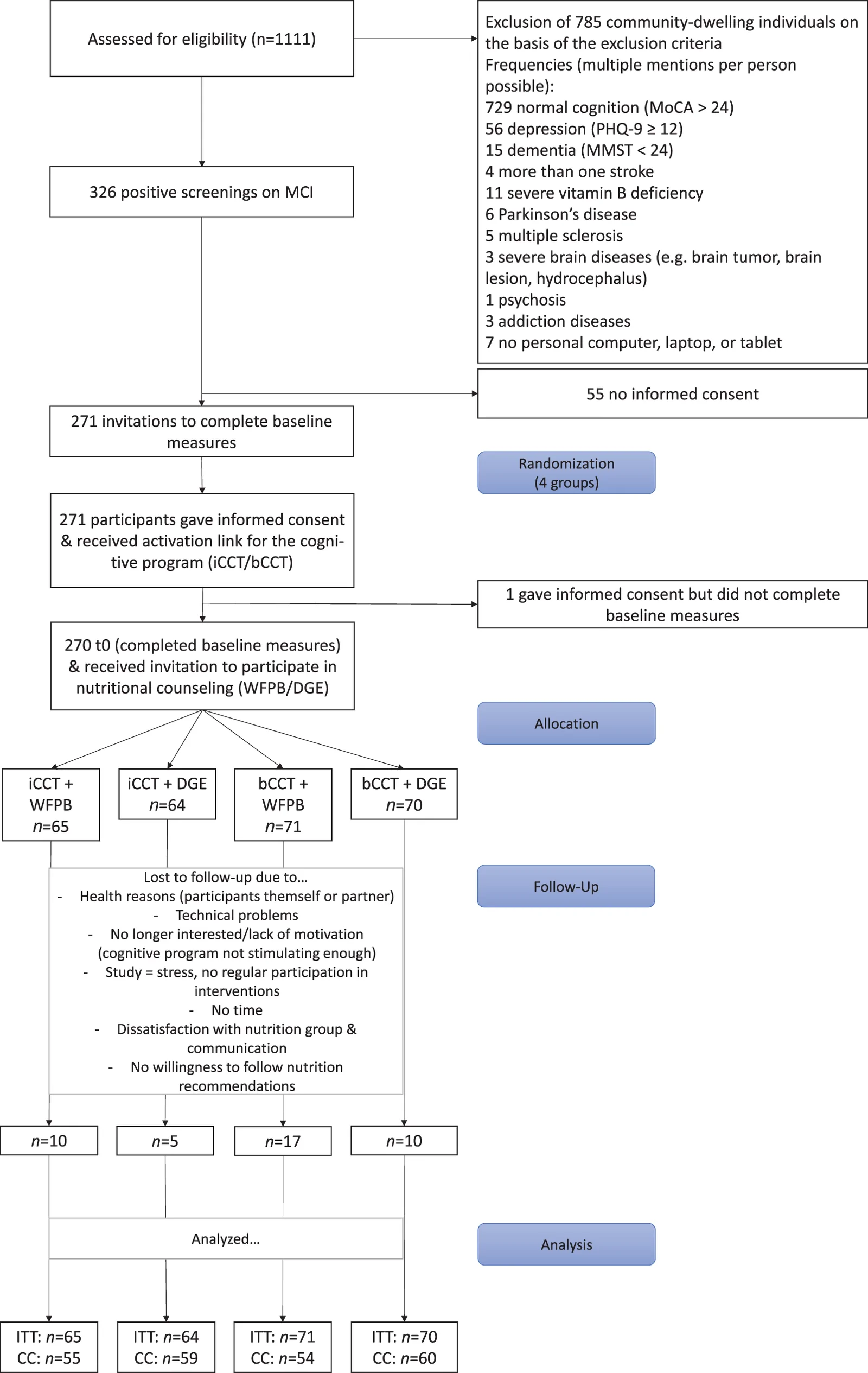
CONSORT flow chart of the BrainFit-nutrition study. *Note*. iCCT: individualized computerized cognitive training; bCCT: basic computerized cognitive training; WFPB: whole-food plant-based nutritional counseling; DGE: nutritional counseling as specified by the German nutrition Society; ITT: intention to treat; CC: complete case

Between January and September 2022, a total of 1,111 people aged 60 or older living at home were screened for eligibility (Fig. 1). About 71% (*n* = 785) were excluded because they did not meet inclusion criteria. The inclusion criteria were met by *N* = 326 individuals, *n* = 271 gave informed consent to take part in the study, were randomized into 19 blocks, and were allocated to the four intervention groups (Fig. 1). Since balance had been achieved in relation to the nutritional intervention before the last randomization, no adjustments were made in the last block. One person did not complete the baseline data and was therefore excluded from the ITT sample (final *n*_*ITT*_ = 270). At the end of the intervention period after six months, *n* = 228 individuals participated in the follow-up assessment (CC sample). The drop-out rate between baseline and t6 was 15.6%.

### Sample characteristics

The CC sample is characterized as follows: They were on average 70.7 years old (Range 60–86 years), and 52% were female (Table 1). Baseline global cognition showed a mean of 22.31 (*SD* = 1.55) on the MoCA scale. According to the International Standard Classification of Education (ISCED [76]), 60% had a high educational level. About half of the participants reported hypertension or hypercholesterolemia. Nevertheless, with an average of 1.08 vascular risk events, the vascular risk score was low in the study sample. The one-year mortality risk was very low in the study population, as 88% (*n* = 201) had Charlson comorbidity scores of 0.

Most of the effect sizes were statistically irrelevant—less than 0.1 for Cramer’s *V* or less than 0.02 for η^2^—except for monthly household net income, smoking, hypertension, weekly cognitive activities, weekly social activities, Charlson comorbidity index, and shared household. There were no significant differences between the sample characteristics of the CC sample and the ITT sample (see Supplementary Material).

### Intervention exposure (adherence)

In Intervention Type I, the iCCT tended to be used more frequently than the bCCT: The iCCT was used for an average of 35.8 min/training day (*SD* = 10.6), 2.95 days/week (*SD* = 1.6), corresponding to approx. 111 min/week. The bCCT was used for an average of 35.6 min/training day (*SD* = 10.8, *p* = 0.432), 2.71 days/week (*SD* = 1.2, *p* = 0.098), corresponding to 105 min/week. However, the bCCT tended to be used over a longer period of the intervention than the iCCT (5.8 months vs. 5.6 months, *p* = 0.262). Therefore, no significant differences could be found between the use of iCCT vs. bCCT.

In Intervention Type II, the WFPB group participated in an average of 7.5 out of 12 online nutritional counseling courses (62.5%, *SD* = 3.6), while the DGE group participated in 7.4 out of 12 courses (61.7%, *SD* = 3.8), with no significant differences between the two versions of this type of intervention (*p* = 0.425).

### Descriptive change in global cognition

In the descriptive analysis, the change in global cognition from t0 to t6 (difference variable t6-t0) was examined in the total sample. The assumptions for performing *t*-tests for dependent samples were fully met: There were no extreme outliers in the data, and a normal distribution could be assumed on the basis of the central limit theorem. Cognitive abilities as captured by the MoCA were significantly higher at t6 (*M* = 24.39, *SD* = 2.77) than at t0 (*M* = 22.31, *SD* = 1.55; 95%-CI (t6-t0): [1.74; 2.43]; *t*(227)=11.96, *p* < 0.001) in the total sample (*n* = 228). This increase (*d* = 0.79) reflects a large effect size according to Cohen [81].

Table 2 provides a summary of the changes in global cognition within each intervention group.

**Table 2:** Course of global cognition within each intervention group at baseline and six-months-follow-up

Group	T0, *M* (*SD*)	T6, *M* (*SD*)	95%-*CI* (t6-t0)	*t*-test	p	d
iCCT+WFPB (*n* = 55)	22.27 (1.55)	24.11 (3.17)	[1.01; 2.66]	4.48	<0.001	0.60
iCCT+DGE (*n* = 59)	22.32 (1.60)	24.68 (2.54)	[1.68; 3.04]	6.95	<0.001	0.90
bCCT+WFPB (*n* = 54)	22.35 (1.62)	24.43 (2.57)	[1.40; 2.74]	6.13	<0.001	0.83
bCCT+DGE (*n* = 60)	22.28 (1.47)	24.35 (2.81)	[1.44; 2.69]	6.59	<0.001	0.88

*Note*. Effect sizes (Cohen’s *d*) as specified by Cohen [81]

### Main analysis (primary outcome)

As part of the main analysis, a 2 × 2 mixed ANCOVA was computed, with time (t0 vs. t6) as the within-subjects factor and intervention type (version of cognitive training [iCCT/bCCT] and version of nutritional counseling [WFPB/DGE]) as the between-subjects factors. Furthermore, seven baseline variables met the criteria to be included as covariates in the model: monthly household net income (η^2^ = 0.02), social activities (η^2^ = 0.02), cognitive activities (η^2^ = 0.03), Charlson comorbidity index (η^2^ = 0.02), smoking (*V* = 0.12), hypertension (*V* = 0.12), and household situation (*V* = 0.12). Baseline MoCA score was included as another variable in the model in order to be able to evaluate time effects (η^2^ = 0.01). The strongest correlation was observed between both adherence factors (*r* = 0.607, *p* < 0.001), so multicollinearity could be excluded.

**Fig. 2:**
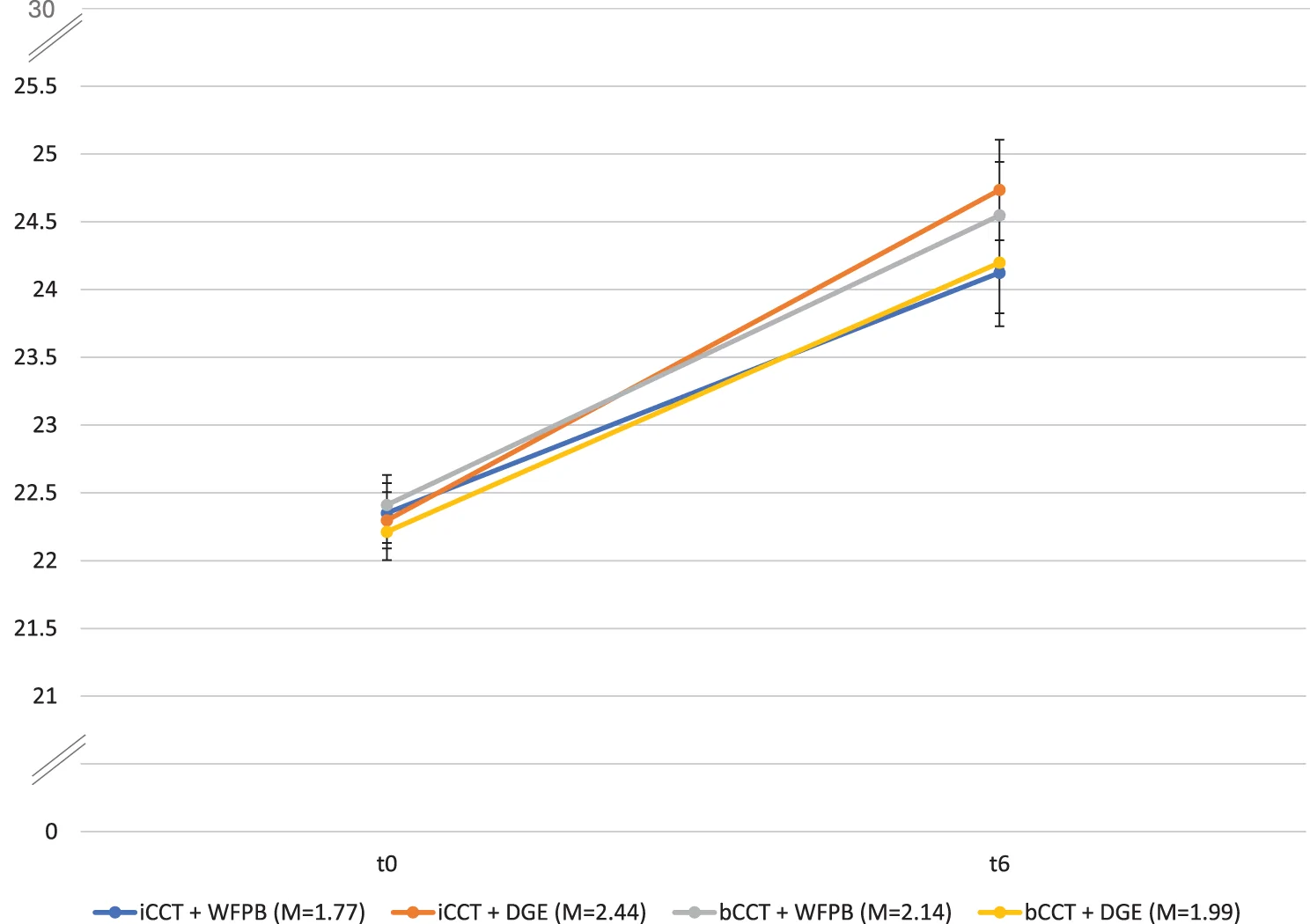
Estimated marginal means and standard errors of the MoCA score in the four intervention groups. *Note*. Estimated marginal means (*Standard Errors*). The mean difference (t6-t0) of the marginal means is indicated in the group legend. Covariates appearing in the model were calculated at the following values: net income: 2602.02, social activities: 10.09, cognitive activities: 18.32, Charlson comorbidity: 0.32, smoking: 0.08, hypertension: 0.45, household situation: 0.69

When using the adjusted means, all four intervention groups showed a significant increase in the global cognition score (MoCA) from baseline to follow-up (*p* < 0.001), with no significant between group differences. Figure 2 shows the estimated marginal means for the group ×time interaction (MoCA score).

The ANCOVA analysis revealed no significant between-group differences for the comparison between the two cognition groups (*p*_cognition_ = 0.891), between the two nutrition groups (*p*_nutrition_ = 0.992), and between the four groups with the specific combination of one cognition and one nutrition intervention (*p*_cognition ×nutrition_ = 0.367; see Table 3). After adjusting for the covariates (Table 3), there was a small but significant main effect of time (*F* = 3.96, *p* = 0.048, partial η^2^ = 0.018). The time ×group interaction was not significant for the version of CCT (iCCT vs. bCCT) or the version of nutritional counseling (WFPB vs. DGE) or across all four intervention groups (*p*_cognition_ = 0.902; *p*_nutrition_ = 0.488; *p*_cognition × nutrition_ = 0.270). The results of the sensitivity analysis (ITT sample) did not significantly differ from those of the main analysis in the CC sample: There was also only a significant main effect of time (see Table 3).

**Table 3:** Results of the 2 × 2 mixed-model ANCOVA examining the between subject effects and the within-subject effects on global cognition (MoCA score) in the CC and ITT sample

		CC sample (*N* = 228)					ITT sample (*N* = 270)			
Effect	F	p	**Partial η**^**2**^	*M*^**~**^**(SE)**	**95%-CI**^**~**^	F	p	**Partial η**^**2**^	*M*^**~**^**(SE)**	**95%-CI**^**~**^
Between subject effects										
Cognition version^#, ^^	0.02	0.891	0.000	0.03 (0.25)	[−0.46; 0.53]	0.02	0.901	0.000	0.03 (0.23)	[−0.42; 0.47]
Nutrition version^●, ^^	0.00	0.992	0.000	−0.01 (0.25)	[−0.50; 0.50]	0.30	0.584	0.001	−0.13 (0.23)	[−0.57; 0.32]
Cognition version × nutrition version^^^^	0.82	0.367	0.003			0.82	0.367	0.003		
iCCT-WFPB				1.77 (0.38)	[1.03; 2.52]				1.69 (0.32)	[1.06; 2.33]
iCCT-DGE				2.44 (0.35)	[1.74; 3.14]				2.36 (0.32)	[1.73; 2.99]
bCCT-WFPB				2.14 (0.38)	[1.39; 2.88]				1.97 (0.31)	[1.37; 2.58]
bCCT-DGE				1.99 (0.36)	[1.28; 2.69]				1.85 (0.31)	[1.25; 2.45]
Within-subject effects										
T^$^	3.96	**0.048**	0.018			7.07	**0.008**	0.027		
T x household net income^+, -^	0.68	0.409	0.003			0.73	0.395	0.003		
T x social activities*	0.16	0.687	0.001			0.20	0.653	0.001		
T x cognitive activities*	0.93	0.337	0.004			0.98	0.323	0.004		
T x Charlson index^§^	0.11	0.738	0.001			0.15	0.702	0.001		
T x smoking^-^	2.26	0.135	0.011			2.05	0.153	0.008		
T x hypertension^-^	0.25	0.621	0.001			0.38	0.538	0.002		
T x shared household^-^	1.21	0.272	0.006			2.13	0.146	0.008		
T x cognition version^#^	0.02	0.902	0.000			0.12	0.726	0.000		
T x nutrition version^●^	0.38	0.488	0.002			0.74	0.391	0.003		
T x cognition version ×nutrition version	1.22	0.270	0.006			1.57	0.212	0.006		

*Note. df* = 1 for all effects; ^**~**^group differences as well as 95% CI are only reported for the between group effects; ^^^the difference between the two groups of each type of intervention (cognition or nutrition); ^^^^ time differences within each intervention combination. ^$^ T: time; ^+^ income per month; ^-^ dichotomous variable; * recorded in hours per week; ^§^ Updated Charlson Comorbidity Index, range: 0–24; ^#^ cognition version: individualized CCT (iCCT) vs. basic CCT (bCCT); ^●^ nutrition version: whole-food plant-based (WFPB) nutrition vs. nutrition recommended by the German Nutrition Society (DGE); significant *p*-values are printed in bold

### Reversion rates

The descriptive analysis of the reversion rates showed that, at baseline, all study participants scored in the MCI range (MoCA score ≤ 24, inclusion criterion). After the 6-months intervention phase, 55% (*n* = 125) of participants left the psychometrically determined MCI range. Within the subgroup of people nearest to the cut-off threshold to cognitive normal at baseline (MoCA t0 = 22–24; *n* = 168), 62% (*n* = 104) reverted to cognitive normal after six months intervention (*M* = 1.91; *SD* = 2.45). For a detailed breakdown by group, see Table 4.

**Table 4:** Reversion rates within the MoCA among the total sample as well as the subsample with high MoCA t0 (22–24)

	iCCT+WFPB, n(%) [95%-CI]^	iCCT+DGE, n(%) [95%-CI]^	bCCT+WFPB, n(%) [95%-CI]^	bCCT+DGE, n(%) [95%-CI]^
Total CC sample (*n* = 228)	26 (47.3) [33.6;60.9]	34 (57.6) [44.6;70.6]	32 (59.3) [45.7;72.8]	33 (55.0) [42.0;68.0]
Subgroup t0-MoCA 22–24 (*n* = 168)	22 (53.7) [37.7;69.6]	27 (61.4) [46.4;76.3]	27 (64.3) [49.2;79.4]	28 (68.3) [53.4; 83.2]

*Note*. CC = complete case – main analysis strategy. ^95%-CI belongs to the percentage rate

There were no significant differences in the reversion rates to MoCA > 24 between the four randomized groups in the total sample (χ^2^ = 1.88; *p* = 0.597) as well as in the subsample MoCA t0 = 22–24 (χ^2^ = 2.00; *p* = 0.573).

### Safety (adverse events)

During the six-months intervention phase in total 55 SAEs and AEs occurred in 45 participants. Eight SAEs appeared in seven study participants (lung cancer, prostatitis, cardiac arrhythmia, falls (2), pain (2), adhesive ileus). All SAEs were classified as not related to the study intervention. Nevertheless, they were reported more often in the cognitive active control groups (bCCT: 6) than in the cognitive intervention groups (iCCT: 2; bCCT-WFPB: 3, bCCT-DGE: 3, iCCT-WFPB: 1, iCCT-DGE: 1).

Further, 34 individuals reported 42 AEs. However, these were not related to the study intervention (e.g., influenza, COVID-19 infection, … ). In addition, five study participants reported an unintentional weight loss of approximately 2–3 kg as a result of changing their diet in accordance with the nutritional recommendations. These AEs occurred mostly in the nutritional intervention group (bCCT-WFPB: 3, bCCT-DGE: 1). This weight loss is not considered clinically critical.

### Research hypothesis

In notable contrast to the hypotheses, no differences were observed between the MCI specified versions and the active control versions within the two types of interventions (primary hypotheses I+II). In particular, the combination of the two MCI specified versions of each type of intervention (iCCT and WFPB) showed no advantage over the other groups (secondary hypothesis). However, there was a significant improvement in global cognition from t0 to t6 in all four intervention groups.

## Discussion

Aim of this RCT was to examine the effects on global cognition of combining two different types of interventions—CCT along with a nutritional counseling. Both were available in an MCI specified version (iCCT or WFPB diet) and an active control version (bCCT or DGE diet). Study participants were randomized to one of the four intervention groups in a 2 × 2 design.

Contrary to the a priori hypotheses, we found no evidence of differential change from the CCT format, dietary counseling format, or their interaction. During the six-months intervention period, global cognition improved by 2.1 points on the MoCA across all intervention groups. This finding is consistent with the multivariate result of a significant main effect of time. Since there were no differences in the therapy dose between the two versions of intervention per intervention type (approx. 110 min CCT, approx. 7.5 of 12 nutritional counseling group sessions), the therapy dose does not need to be taken into account when comparing the results between the groups. Both types of interventions appear to be safe, as no SAEs related to the study interventions were reported. From a pragmatic implementation perspective, the similar cognitive outcomes across all four active intervention arms suggest that less complex (and potentially better scalable) intervention formats may warrant further evaluation as options for low-threshold community-based MCI management. However, this trial was designed as a superiority trial and did not include a pre-specified equivalence or non-inferiority margin; therefore, the absence of significant between-group differences cannot be interpreted as evidence of equivalent effectiveness. Future pragmatic, non-inferiority and larger scale clinical trials including health-economic studies are warranted to examine whether lower-complexity or less restrictive intervention formats such as bCCT and DGE-based counseling may provide comparable clinical benefit with lower implementation burden and barriers.

Due to the recruitment method and the digital requirements for participation, the educational level of most study participants was above average, which is not unusual in intervention studies using digital tools with older people with MCI [23]. Participants showed a high activation level at baseline in the three areas in which cognitive decline can be prevented: cognitive, physical, and social activities. Therefore, the sample was likely highly motivated to prospectively minimize the limitations they experienced from MCI, in the sense of a self-optimization process. This introduces bias and may limit the effects that can be achieved from additional interventions - in the sense of a ceiling effect - and may have rendered group differences less likely to occur.

### Comparison of cognition-related results with the pilot study

In contrast to the pilot study [32], whose study design was expanded in the present RCT to include online nutritional counseling as one type of intervention, no significant group differences revealed in the present study. Compared with the pilot study, two findings emerged: (a) The average increase of 2.1 points in the MoCA score (within six months) from using the iCCT program—in combination with an online nutritional counseling—was roughly the same as in the pilot study (2.0 points). (b) The same increase of an average of 2.1 points in the MoCA score in six months when using the bCCT was about twice as large as in the pilot study (0.9 points). It is possible that combining the cognitive therapy with an online nutritional counseling boosted the effect of the bCCT in this sample of educated, motivated study participants so that the increase in the MoCA score was just as large as under the iCCT, which was also combined with an online nutrition program.

Compared with the pilot study [32], the improvement in the present study cannot be attributed to any specific combination of the two types of interventions. Thus, combining the cognitive intervention with the second intervention component (nutritional counseling) likely may have resulted in a more general lifestyle change for the participants.

### Learning effects and spontaneous remission

The study attempted to avoid learning effects as much as possible in relation to the primary endpoint of general cognitive performance, as measured by the MoCA: a six-months intervention period was between the two measurement points, a time period that reduces learning effects in individuals with confirmed amnestic MCI [85], and a parallel version of the MoCA was used in the present study at t6. Nevertheless, the possibility of a learning effect cannot be completely ignored [86]. Furthermore, it should also be taken into account that no performance test is 100% reliable. In healthy older adults the reliable change index of the MoCA has a range of four points over 12 months [87]. However, this “noise” due to measurement uncertainty does not account for systematic changes over time, since reliability limitations occur at every measurement point. Also, it cannot be excluded that the statistical artifact of regression toward the mean may have influenced the change in cognitive performance in the MCI sample [88].

There are varying results regarding spontaneous remissions in MCI, as remission rates across different studies range from 5% [89] to 50% [90]. Most studies report a rate of around 12% to 20% [91–93]. According to Canevelli et al. [94], these differences, with 8% in clinical-based studies and 25% in population-based studies, can also be attributed to the study setting. Yu et al. [93] reported a remission rate of 18%, such that reverters were younger than stable MCI patients and differed in terms of clinical parameters. Compared with individuals with stable normal cognition, MCI reverters were older, tended to be female, had fewer years of schooling, and differed in terms of various clinical parameters. In addition, the cut-off value of ≤ 24 points on the MoCA used in the present study can be assumed to have a specificity of around 80% [69], which corresponds to a false-positive rate of about 20%. In the present study, however, the remission rate to psychometrically recorded, cognitively unremarkable values in all four groups was 45% to 59%, which is significantly higher than the average spontaneous remission rates between 8 and 25% in a recent review and meta-analysis [94] of up to 20% reported in the literature. The higher reversion rate within the subgroup MoCA 22–24 could be explained by the fact that these individuals were closer to the cut-off point for cognitive normal than individuals with more severe cognitive impairments. From a clinical perspective, MCI represents a subjective and objective decline in cognition and function associated with an increased risk of disease progression [95]. Thus, an increase in cognitive impairment must be expected in the majority of cases. However, causal inference is substantially limited in the current study due to the absence of a non-intervention control group. Therefore, the reversion rate to “cognitive normal” resulting from the interventions in this study should be interpreted as a trend.

### Comparison with other CCT results in the literature

Cognitive training, especially in its computerized form, has been shown to be an effective intervention for improving cognitive abilities in people with MCI [23, 26, 29]. Furthermore, cognitive training appears to be superior to other non-pharmacological interventions in terms of its effect on cognition [22]. However, since the different types of interventions examined in this study were previously studied in separate groups of participants, whereas each group of participants received a combined intervention in our study, the results are not comparable. In contrast to Tsiakiri et al. [9], we found a significant improvement in global cognition (measured by the MoCA) in the present study when cognitive training was combined with nutritional counseling. Tsiakiri et al., on the other hand, only found an effect in terms of maintaining cognitive abilities, measured with the Cambridge Cognitive Examination, in the intervention group. The non-intervention group, on the other hand, showed cognitive decline. Thus, the present CCT seems to have worked better for our participants than in the study by Tsiakiri et al. [9]. One reason for this finding could be that both versions of CCT in the present study provided a good user experience [60]. Such positive experiences are important for maintaining motivation and frequency of use on the part of users, thereby contributing to long-term training success [27]. However, when interpreting the results comparatively, it is necessary to acknowledge that comparability is limited because numerous different cognitive parameters were measured in the various studies [96].

### Comparison with results from digital nutrition programs for individuals with MCI

Several factors may explain why no significant differences were observed between the WFPB and the DGE counseling conditions. First, the DGE-based program represents a health-promoting dietary pattern itself and therefore constitutes an active control condition, potentially resulting in a ceiling effect. Second, dietary behavior was assessed in a secondary analysis of the BF-N study [61], indicating that dietary changes occurred in both groups and that the overall contrast between the counseling formats remained modest. Third, an intervention period of six months may still be insufficient for dietary changes to translate into measurable differences in cognitive outcomes, particularly when assessed with screening instruments such as the MoCA. Specifically, the secondary dietary data analysis [61] found that while both groups improved dietary quality scores from baseline, the between-group difference in achieved dietary pattern remained small, reflecting the high standard of the DGE active comparator. The DGE-based program represented an active, evidence-based healthy-diet comparator rather than a passive or usual-diet control. This strong active comparator may have reduced the likelihood of detecting differential cognitive effects between the nutritional counseling arms and should be considered when interpreting the results.

Several nutritional studies and systematic reviews have suggested that dietary factors can influence cognitive performance in individuals with MCI and related disorders. For example, systematic evidence indicates that antioxidant-rich foods, omega-3 fatty acid supplementation, and multinutrient approaches may improve specific cognitive domains and cognition-related biomarkers in MCI, although effects on global cognition are inconsistent across studies [97]. Despite these indications of benefits, the current literature also highlights methodological limitations, including small sample sizes, heterogeneous intervention designs, and variability in cognitive outcome measures, which complicate definitive conclusions about the magnitude and consistency of diet-related effects on cognition [98].

Although digital nutritional interventions specifically targeting individuals with MCI remain scarce, the emerging literature has highlighted both the potential and the gaps in this domain. A recent protocol for a smartphone-based multidomain digital therapeutic that includes dietary planning demonstrated the feasibility of integrating nutrition into mobile interventions for MCI, though outcome data are not yet available [99].

Taken together, these findings suggest that while integrating nutritional counseling into digital interventions for MCI is conceptually promising, there is presently limited empirical evidence on cognitive outcomes from fully digitalized dietary programs. Future research from methodologically rigorous trials with cognitive endpoints is needed to clarify whether remote diet counseling or digital nutritional support tools can reliably influence cognitive trajectories in people with MCI.

### Comparison with other multi-domain interventions

The literature shows, that multidomain interventions are superior to single-domain interventions [17, 19, 100, 101]. In particular, the combination of cognitive training and physical activity has proven to be effective, although they also did not find a significant group ×time interaction [101]. The combination of CCT and nutritional counseling in the previous study showed an improvement in cognitive abilities across all groups, comparable to the improvement observed in the intervention group of the single-domain cognitive based pilot study [32]. Therefore, while synergistic effects resulting from the combination of cognitive and nutrition-related measures in the active control interventions are plausible, they have not yet been demonstrated. In contrast to Choi et al. [102], the intensity of the active control group within the BrainFit-Nutrition study was comparable to the MCI-specific interventions.

Further, the results are also in line with the multidomain intervention study of Xu et al. [18], finding also no significant differences between groups, but significant effects over time in all three groups. Possible reasons for the lack of group differences include a trend toward the middle, natural progression of MCI, or additional interventions that were not recorded. This aspect could also be important for interpreting the results of this study, as the MCI individuals in this study were highly cognitively, physically, and socially active in their everyday lives.

### Limitations

For ethical reasons, and on the basis of the results of the pilot study, we decided not to use a “real” control group. Therefore, it is not possible to compare the effectiveness of the interventions with an untreated course. The nutritional intervention consisted only of online nutritional group counseling; participants were free to decide whether and to what extent they would implement the recommendations. Since no personal data on participants’ use of the CCTs were transmitted, we had no objective data on the use of the CCTs. The training intensities reported in this study were based on subjective information with potential sources of bias that this subjectivity entails. Furthermore, the primary outcome was assessed with a screening instrument (MoCA) and when interpreting the results of this performance test, potential learning effects – even though parallel tests were used in the follow-up measurement of this study – and limitations in reliability must be taken into account. It might be possible to identify meaningful group differences by using extensive test batteries. Also, the inclusion criteria did not fully cover the clinical diagnostic criteria for MCI. Due to the recruitment process and, in particular, the minimum technical requirements for participation in the study, the sample had an above-average level of education, which could further limit the representativeness of the results. In addition, selection effects may have occurred due to the recruitment and willingness of participants to take part in two different types of interventions, meaning that more health-conscious individuals tended to participate in the study, thereby obscuring potential group differences. It is also important to mention at this point that, despite the innovative study design and the chosen data analysis strategy, due to the combination of CCT and nutritional counseling in all four study arms, ultimately no reliable statements about the effectiveness of the individual intervention components can be made.

Furthermore, the present manuscript does not include detailed dietary intake outcomes as part of the primary outcome analysis. Adherence to the nutritional intervention was operationalized as session attendance, which reflects counseling-format engagement but does not directly capture actual and individual dietary change. Dietary behavior was assessed using food-frequency questionnaires and has been reported in a pre-planned secondary analysis [61]. This analysis found that while both groups improved dietary patterns from baseline, the between-group contrast in actual dietary intake remained modest. This modest dietary contrast, combined with the fact that the DGE comparator constituted an active, evidence-based healthy-diet condition rather than a passive control, provides a plausible mechanistic explanation for the absence of significant differential cognitive outcomes. Future trials in this area should focus on including validated dietary assessment instruments and nutritional biomarkers as adherence or mechanistic measures alongside session attendance and patient reported outcome measures.

## Conclusion

In conclusion, a combined remote program of CCT and online nutritional counseling was associated with significant improvement in MoCA scores over six months in individuals with MCI in all four study groups. Contrary to the primary hypotheses, the more MCI specified interventions did not outperform the basic variants of CCT and dietary counseling. The interaction of the two MCI specified versions of the interventions also showed no benefits. These findings suggest that, in a digitally literate and highly motivated MCI cohort, structured participation in a multimodal remote lifestyle program may be more relevant to short-term changes in MoCA scores than assignment to specific intervention conditions.

### Future research

Based on Inui et al. [1], longer follow-up times covering a period up to five years are particularly valuable. Such data are currently being collected, beginning with a 12 months follow-up, followed by testing every year, and will be published later. Future trials should also include usual-care comparators, a separate analysis of the two intervention types, objective adherence and use measures, as well as biological endpoints to clarify mechanisms, sustainability of effects, and clinical relevance.

## Electronic supplementary material

Below is the link to the electronic supplementary material.

Supplementary Material

## Data Availability

The data sets used and/or analyzed for the current study are available from the corresponding author upon reasonable request. The data are not publicly available due to privacy.

## Declarations

### Cognitive tests

The MMSE licenses were purchased from Hogrefe. Permission to use the MoCA was obtained.

### AI-assisted writing (nutritional parts)

Artificial intelligence (AI) tools (Claude Code) were used in the preparation of the nutritional parts of this manuscript in a limited and supervised capacity. Specifically, AI-assisted summarization was employed to support the synthesis of relevant literature. Also, text optimization of few selected passages was AI-supported to a very limited extend. All AI-generated or AI-assisted content was critically reviewed, edited, and approved by the authors. The authors take full responsibility for the accuracy, integrity, and intellectual content of the final manuscript. AI tools were not used for data analysis, conceptualization, program development, interpretation of results, or scientific decision-making. This applies only to the sections on nutritional aspects, but not to the rest of the manuscript.

## Abbreviations

AE: Adverse event
AI: Artificial intelligence
bCCT: Basic computerized cognitive training
CCT: Computerized cognitive training
CCTB: Computerized cognitive training battery
DASH Diet: Dietary Approaches to Stop Hypertension
DGE: German Nutrition Society (Deutsche Gesellschaft für Ernährung)
iCCT: Individualized computerized cognitive training
ISCED: International Standard Classification of Education
MCI: Mild cognitive impairment
MedDiet: Mediterranean Diet
MIND Diet: Mediterranean-DASH Intervention for Neurodegenerative Delay
MMSE: Mini-Mental State Examination
MoCA: Montreal Cognitive Assessment
RCT: Randomized controlled trial
SAE: Severe adverse event
WFPB: Whole-food plant-based diet
