# Supplementary material for: Computerised cognitive training combined with nutritional counseling in mild cognitive impairment - results of a 2 × 2 factorial randomized controlled trial (BrainFit-nutrition study)
Source: Alzheimers Res Ther. 2026 Nov 9;18:247. doi: 10.1186/s13195-026-02255-6 (PMC13753266; doi:10.1186/s13195-026-02255-6)
Supplement: Supplementary file 1 [file 13195_2026_2255_MOESM1_ESM.docx]

**Table 1.** Baseline characteristics of the study participants

| **Variables** | **iCCT+WFPB**  **(*n* = 65)** | **iCCT+DGE**  **(*n* = 64)** | **bCCT+WFPB**  **(*n* = 71)** | **bCCT+DGE**  **(*n* = 70)** | ***p*** | **Group**  **difference**  **(│effect size│)** | **Total**  **(*n* = 270)** | |
| --- | --- | --- | --- | --- | --- | --- | --- | --- |
| **Sociodemographics** |  |  |  |  |  |  |  | |
| Age (years), *M* (*SD*) | 71.03 (6.5) | 70.61 (6.6) | 70.68 (6.2) | 70.94 (8.03) | .982 | 0.01^$^ | 70.81 (6.9) | |
| Sex (female), *n* (%) | 35 (53.8) | 33 (51.5) | 37 (52.1) | 36 (51.4) | .992 | 0.02^#^ | 141 (52.2) | |
| Education level (ISCED)^1^, *n* (%) |  |  |  |  | .626 | 0.08^#^ |  | |
| Low/middle | 28 (43.1) | 24 (37.5) | 33 (46.5) | 26 (37.1) |  |  | 104 (38.5) | |
| High | 37 (56.9) | 40 (62.5) | 38 (53.5) | 44 (62.9) |  |  | 159 (58.9) | |
| Household net income per month (Euro), *M* (*SD*) | 2662 (687) | 2464 (836) | 2617 (689) | 2677 (643) | .313 | 0.02^$^ | 2607 (716) | |
| Employment (yes), *n* (%) | 18 (27.7) | 19 (29.6) | 17 (23.9) | 22 (31.4) | .783 | 0.06^#^ | 76 (28.1) | |
| Other persons living in the household (yes), *n* (%) | 47 (72.3) | 42 (65.6) | 54 (76.1) | 47 (67.1) | .520 | 0.09^#^ | 190 (70.4) | |
| MoCA Baseline, *M* (*SD*) | 22.28 (1.56) | 22.38 (1.56) | 22.35 (1.73) | 22.41 (1.48) | .966 | 0.01^$^ | 22.36 (1.58) | |
| MoCA t6, *M* (*SD*) | 24.02 (2.99) | 24.68 (2.45) | 24.31 (2.47) | 24.30 (2.69) | .576 | 0.01^$^ | 24.33 (2.65) | |
| **Risk factors for cognitive decline** | | | | | | | |  |
| Depressiveness (PHQ-9^2^), *M* (*SD*) | 3.8 (2.9) | 3.8 (2.7) | 3.6 (2.7) | 3.9 (3.2) | .908 | 0.01^$^ | 3.8 (2.9) | |
| Vascular risk (sum score^3^), *M* (*SD*) | 0.97 (0.83) | 1.1 (0.87) | 1.04 (0.86) | 1.11 (0.89) | .773 | 0.01^$^ | 1.06 (0.86) | |
| Smoking (last 30 days, yes), *n* (%) | 6 (9.2) | 2 (3.1) | 4 (5.6) | 3 (4.3) | .802 | 0.08^#^ | 15 (5.6) | |
| Hypertension (yes), *n* (%) | 28 (43.1) | 30 (46.9) | 32 (45.1) | 35 (50.0) | .871 | 0.05^#^ | 125 (46.3) | |
| Hypercholesterolaemia (yes), *n* (%) | 26 (40.0) | 31 (48.4) | 30 (42.3) | 30 (42.9) | .798 | 0.06^#^ | 117 (43.3) | |
| Diabetes (yes), *n* (%) | 3 (4.6) | 7 (10.9) | 7 (9.9) | 10 (14.3) | .310 | 0.12^#^ | 27 (10.0) | |
| Medication score^4^, *M* (*SD*) | -0.08 (0.37) | -0.06 (0.24) | -0.13 (0.56) | -0.07 (.0.31) | .770 | 0.01^$^ | -0.1 (0.39) | |
| Cognitive activities (hours per week), *M* (*SD*) | 16.1 (11.2) | 17.7 (9.2) | 18.5 (14.4) | 20.8 (20.9) | .334 | 0.01^$^ | 18.3 (14.8) | |
| Physical activities (hours per week), *M* (*SD*) | 10.3 (7.1) | 11.8 (9.8) | 11.1 (10.8) | 10.6 (8.1) | .776 | 0.01^$^ | 10.9 (9.0) | |
| Social activities (hours per week), *M* (*SD*) | 8.5 (6.5) | 11.6 (9.8) | 9.2 (7.4) | 10.6 (7.9) | .110 | 0.02^$^ | 10.0 (8.0) | |
| Charlson Index^5^, *M* (*SD*) | 0.14 (0.39) | 0.34 (0.74) | 0.37 (0.8) | 0.36 (0.7) | .167 | 0.02^$^ | 0.3 (0.7) | |
| Body Mass Index, normal (≥ 19 and ≤ 25)^6^ (yes), *n* (%) | 58 (89.2) | 56 (87.5) | 59 (83.1) | 60 (85.7) | .756 | 0.07^#^ | 233 (86.3) | |
| **Interventions** |  |  |  |  |  |  |  | |
| Adherence to nutritional counseling^7^, *M* (*SD*) | 6.4 (4.0) | 7.1 (4.3) | 6.4 (4.0) | 6.5 (4.1) | .764 | 0.01^$^ | 6.6 (4.1) | |
| Fully adherence to nutritional counseling ^8^, *n* (%) | 11 (16.9) | 13 (20.3) | 13 (18.3) | 11 (15.7) | .912 | 0.04^#^ | 48 (17.8) | |
| Adherence to CCTB (months) ^9^, *M* (*SD*) | 4.9 (2.6) | 5.5 (2.4) | 4.8 (2.7) | 5.0 (2.5) | .486 | 0.01^$^ | 5.1 (2.5) | |
| Fully adherence to CCTB ^8^, *n* (%) | 39 (60.9) | 46 (71.9) | 45 (60.7) | 46 (65.7) | .543 | 0.09^#^ | 176 (65.2) | |

*Note*. ^$^ η²; ^#^ Cramer’s V; iCCT: individualized computerized cognitive training; bCCT: basic computerized cognitive training; WFPB: whole-food plant-based nutritional counseling; DGE: nutritional counseling as recommended by the German Nutrition Society; ^1^ In the low education category, there were only n=7 cases in three groups as determined by the ISCED (International Standard Classification of Education), so these cases were combined with the medium ISCED category; ^2^ Patient Health Questionnaire 9; ^3^ Number of vascular risk factors: range 0 (no vascular risk factor) to 4 (four vascular risk factors); ^4^ Sedating (< 0) or stimulating (> 0) effect or side effect of the prescribed medications: sum score for each medication, which was ranked from -2 (strong sedating effect) to +2 (strong stimulating effect); ^5^ Updated Charlson Comorbidity Index: range: 0–24; ^6^ normal range of the Body Mass Index; ^7^ adherence captured through the number of counseling sessions attended; ^8^number of participants attending each nutritional counseling session or every CCTB (Computerized Cognitive Test Battery, Graessel et al., 2024b) upload; ^9^ number of months with active upload of the CCTB.
